# Bone Microarchitecture and Strength in Long‐Standing Type 1 Diabetes

**DOI:** 10.1002/jbmr.4517

**Published:** 2022-03-08

**Authors:** Lilian Sewing, Laura Potasso, Sandra Baumann, Denis Schenk, Furkan Gazozcu, Kurt Lippuner, Marius Kraenzlin, Philippe Zysset, Christian Meier

**Affiliations:** ^1^ Department of Endocrinology Diabetology and Metabolism University Hospital Basel Basel Switzerland; ^2^ Department of Clinical Research University of Basel Basel Switzerland; ^3^ ARTORG Center, University of Bern Bern Switzerland; ^4^ Department of Osteoporosis University Hospital Bern Bern Switzerland; ^5^ Endocrine Clinic and Laboratory Basel Switzerland

**Keywords:** TYPE 1 DIABETES, BONE TURNOVER, BMD, BONE STRENGTH, BONE MICROARCHITECTURE

## Abstract

Type 1 diabetes (T1DM) is associated with an increased fracture risk, specifically at nonvertebral sites. The influence of glycemic control and microvascular disease on skeletal health in long‐standing T1DM remains largely unknown. We aimed to assess areal (aBMD) and volumetric bone mineral density (vBMD), bone microarchitecture, bone turnover, and estimated bone strength in patients with long‐standing T1DM, defined as disease duration ≥25 years. We recruited 59 patients with T1DM (disease duration 37.7 ± 9.0 years; age 59.9 ± 9.9 years.; body mass index [BMI] 25.5 ± 3.7 kg/m^2^; 5‐year median glycated hemoglobin [HbA1c] 7.1% [IQR 6.82–7.40]) and 77 nondiabetic controls. Dual‐energy X‐ray absorptiometry (DXA), high‐resolution peripheral quantitative computed tomography (HRpQCT) at the ultradistal radius and tibia, and biochemical markers of bone turnover were assessed. Group comparisons were performed after adjustment for age, gender, and BMI. Patients with T1DM had lower aBMD at the hip (*p* < 0.001), distal radius (*p* = 0.01), lumbar spine (*p* = 0.04), and femoral neck (*p* = 0.05) as compared to controls. Cross‐linked C‐telopeptide (CTX), a marker of bone resorption, was significantly lower in T1DM (*p* = 0.005). At the distal radius there were no significant differences in vBMD and bone microarchitecture between both groups. In contrast, patients with T1DM had lower cortical thickness (estimate [95% confidence interval]: −0.14 [−0.24, −0.05], *p* < 0.01) and lower cortical vBMD (−28.66 [−54.38, −2.93], *p* = 0.03) at the ultradistal tibia. Bone strength and bone stiffness at the tibia, determined by homogenized finite element modeling, were significantly reduced in T1DM compared to controls. Both the altered cortical microarchitecture and decreased bone strength and stiffness were dependent on the presence of diabetic peripheral neuropathy. In addition to a reduced aBMD and decreased bone resorption, long‐standing, well‐controlled T1DM is associated with a cortical bone deficit at the ultradistal tibia with reduced bone strength and stiffness. Diabetic neuropathy was found to be a determinant of cortical bone structure and bone strength at the tibia, potentially contributing to the increased nonvertebral fracture risk. © 2022 The Authors. *Journal of Bone and Mineral Research* published by Wiley Periodicals LLC on behalf of American Society for Bone and Mineral Research (ASBMR).

## Introduction

Type 1 diabetes (T1DM) is known to be associated with an increased risk of hip and nonvertebral fractures.^(^ ^1^, ^2^, ^3^, ^4^, ^5^, ^6^ ^)^ Meta‐analyses have identified an up to sevenfold increase in hip fractures in patients with T1DM aged between 20 and 60 years.^(^ ^1^, ^4^, ^7^ ^)^ In line with this, hip fractures tend to occur 10 to 15 years earlier in patients with T1DM compared with the nondiabetic population.^(^ ^8^, ^9^ ^)^ Regarding vertebral fractures there is less evidence available but some studies point to an increased risk of vertebral fractures in patients with T1DM.^(^ ^10^, ^11^, ^12^ ^)^

The fracture risk in T1DM is accompanied by a reduction in areal BMD (aBMD), particularly at the hip,^(^ ^7^, ^13^ ^)^ which is apparent in both male and female patients.^(^ ^14^ ^)^ However, the modest reduction in aBMD does not explain the extent of fracture risk. Vestergaard^(^ ^7^ ^)^ showed that, based on aBMD, the estimated fracture risk in T1DM is only 1.4‐fold higher than in controls. In addition to a reduced aBMD, alterations in bone quality characterized by variations in bone remodeling rates as well as changes in bone microarchitecture may represent an important determinant of diabetes‐related bone fragility. High‐resolution quantitative computed tomography at peripheral sites (HRpQCT) allows to quantitatively assess volumetric bone mineral density (vBMD), bone geometry, and microarchitecture in a compartmental fashion with separate analyses of trabecular and cortical bone compartments.^(^ ^15^ ^)^ HRpQCT data on bone microarchitecture have been mainly obtained for type 2 diabetes (T2DM)^(^ ^16^, ^17^, ^18^ ^)^; there are only a few data on patients with T1DM. So far, the use of HRpQCT in T1DM revealed mainly differences in the trabecular compartment and trends of higher cortical porosity as compared to nondiabetic controls.^(^ ^19^, ^20^, ^21^ ^)^

Nowadays life expectancy in patients with T1DM is gradually increasing^(^ ^19^ ^)^ and more patients will survive long enough to develop fractures. Long exposure to the disease is considered to be an independent risk factor for fractures.^(^ ^22^, ^23^ ^)^

There has been some debate as to whether the presence of microvascular complications in T1DM might impact bone microarchitecture and influence fracture risk. In support of this notion, Shanbhogue and colleagues^(^ ^20^ ^)^ found an altered bone microarchitecture in cortical and trabecular compartments in patients with T1DM and microvascular disease only. A recent study showed a higher cortical porosity in type I diabetics with diabetic neuropathy compared to patients without neuropathy.^(^ ^24^ ^)^ However, most studies investigating bone quality in T1DM were performed in patients with relatively short disease duration.^(^ ^25^ ^)^ Maddaloni and colleagues^(^ ^26^ ^)^ examined bone health in patients with long‐standing T1DM (mean diabetes duration, 52 years) and high rates of microvascular complications, but they did not investigate bone microarchitecture.

Within the present study we aimed (i) to examine the effects of long‐standing T1DM (disease duration ≥25 years) on densitometric, microstructural, biochemical and estimated biomechanical bone properties; and (ii) to assess whether microangiopathy, a long‐term diabetic complication, and specifically diabetic neuropathy, has an independent effect on bone microstructure.

## Patients and Methods

### Study population

This is a single‐center, cross‐sectional, case‐controlled study. Patients with long‐standing T1DM and nondiabetic controls were recruited from the Endocrine Clinic at University Hospital Basel, Switzerland, as well as via press advertisement. The study size of at least 57 subjects per group to reach a given power of 90% was calculated for the comparison of total vBMD according to data from Shanbhogue and colleagues.^(^ ^20^ ^)^ Subjects were eligible for inclusion if they had type 1 diabetes with a disease duration of at least 25 years with or without microvascular disease. We excluded patients with coexisting metabolic bone disease, a history of osteoporosis, or medical conditions affecting bone health (eg, hepatic [serum aspartate aminotransferase {AST} more than three times the upper limit of normal] or renal insufficiency [chronic kidney disease stage IV and V], metastatic bone disease, inflammatory bowel disease, thyrotoxicosis, celiac disease, hypogonadism, hypercortisolism).

Data on comorbidities, microvascular and macrovascular disease, medication use, historical glycated hemoglobin (HbA1c) levels (2, 5, 7, and 10 years before enrollment), menopausal status, smoking status, alcohol intake, calcium and vitamin D intake, fracture history, family history regarding hip fractures, and falls were obtained during the study visit and from past medical records. Height and weight were measured on site. We assessed lower extremity strength, balance, and gait by performing timed up and go test (time in seconds to rise from an armchair, walk 3 m, turn around 180 degrees, walk back to the chair, and sit down again) and chair‐rising test (minimum time in seconds to complete five cycles of rising from a standard chair until standing fully erect and sitting down again with the arms folded across the chest).^(^ ^27^ ^)^ Fracture Risk Assessment Tool (FRAX) score was calculated using the online fracture risk assessment tool for Switzerland provided by the Centre for Metabolic Bone Diseases at Sheffield University, UK.

### Assessment of microvascular complications

The presence of diabetic neuropathy (distal symmetric polyneuropathy) was defined by vibration perception test using a 128‐Hz Riedel Seiffer tuning fork at the first metatarsophalangeal joint (grade ≤4/8 in patients >60 years and <6/8 in patients ≤60 years indicating clinical neuropathy according to manufacturer guideline). Data on diabetic retinopathy and/or diabetic nephropathy (presence of urinary albumin creatinine ratio >30 mg/g in a random voided urine sample when ≥2/3 tests were positive) were obtained from past medical records and by interview.

### Biochemical assessment

Fasting blood samples were drawn between 8:00 a.m. and 11:00 a.m. After analysis for HbA1c (Alere Afinion; Abbott, Chicago, IL, USA) and fasting glucose, serum samples were stored at −20°C until analysis (analysis within 12 months). Samples were analyzed for calcium, phosphate, 25OH vitamin D, creatinine, urinary calcium, and urinary creatinine by standard method on an autoanalyzer (Cobas Integra 400plus; Roche Diagnostics, Basel, Switzerland). Procollagen type 1 N propeptide (P1NP), beta‐CrossLaps (CTX), intact parathyroid hormone (iPTH), and 25‐Hydroxyvitamin D (25OHD) were assessed in serum by electrochemiluminescence immunoassays (ECLIA) (Cobas® e411 autoanalyzer; Roche Diagnostics, Rotkreuz, Switzerland). The intraassay and interassay variation was 2.0% to 8.4% for CTX, 1.2% to 3.3% for P1NP, 2.2% to 10.7% for 25OHD, and 1.2% to 2.0% for iPTH, respectively. Serum bone‐specific alkaline phosphatase (BAP) was measured by ELISA (MicroVueBAP; Quidel, San Diego, CA, USA) with an intraassay variation of <5.8% and an interassay variation of 7.6%.

### aBMD, trabecular bone score, and vertebral fracture assessment

We assessed aBMD at the lumbar spine, hip, and distal radius by dual‐energy X‐ray absorptiometry (DXA) using a Hologic Discovery densitometer (Horizon A, S/N 200174); Hologic, Bedford, MA; USA). Short‐term precision of the densitometer was determined by performing duplicate scans in 20 patients. The following coefficients of variation were calculated: 1.1% (spine), 1.4% (femoral neck), 1.9% (trochanteric region), and 1.1% (total hip). Device quality assurance assessments and regular machine calibrations were performed and monitored according to the manufacturer's recommendations. Every DXA scan was assessed by the investigator for additional quality control; vertebrae containing foreign material or showing degenerative changes were excluded from aBMD calculation.

TBS iNsight Imaging Software (version 1,8; Med‐Imaps, Pessac, France) was used to compute trabecular bone score (TBS) from spine DXA scans.

To screen for prevalent vertebral fractures, we performed vertebral fracture assessment (VFA) on lateral spine scans that were generated alongside DXA scans.

### vBMD, bone microarchitecture, and bone strength

#### Scanning

**Fig. 1:**
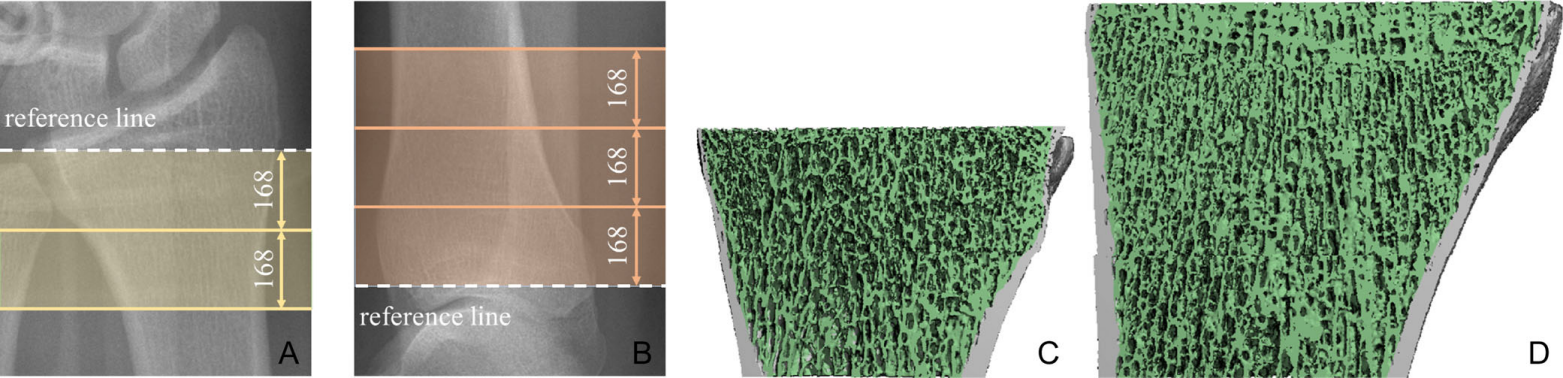
Reference line position on scout view images and qualitative visualization of multiple sections. (*A*) Distal radius: reference line position at the dense articular surface, formed with the scaphoid and lunate fossae of the radiocarpal joint. Scan region: adjacent double section (2 × 168 = 336 slices) without offset. (*B*) Distal tibia: reference line position at the proximal margin of the dense structure formed by the tibial plafond. Scan region: adjacent proximal triple section (3 × 168 = 504 slices) without offset. (*A*,*B*) from^(^ ^29^ ^)^; (*C*) 3D image of the segmented radial double section; (*D*) 3D image of the segmented tibial triple section.

Subjects were evaluated at the department of osteoporosis, University Hospital of Bern (Inselspital) using an HRpQCT scanner (XtremeCT II; Scanco Medical, Brüttisellen, Switzerland) with standard settings for in vivo measurements (60 kVp, 900 μA, 100 ms integration time) and at an isotropic voxel size of 60.7 μm. A calibration phantom was measured for daily and weekly quality control according to the manufacturer's protocol. We fixed the nondominant forearm and equilateral leg of each subject in a carbon fiber cast provided by the scanner manufacturer. In case of a previous distal radius or distal tibia fracture the nonfractured side was used: In eight patients with T1DM and 11 controls the nonfractured dominant side was evaluated. Following positioning, the reference line was set on a standard scout view image according to Bonaretti and colleagues.^(^ ^28^, ^29^ ^)^ For the radius, the reference line was placed at the proximal boundary of the compact articular surface formed with the radiocarpal joint (lunate fossae and scaphoid). Without an offset the adjoining double section was measured (336 slices, 20.4 mm). At the distal tibia the operator placed the reference line at the proximal boundary of the compact structure formed by the tibia plafond; the adjoining triple section (504 slices, 30.6 mm) was measured. The reference lines and respective measured regions are shown in Fig. 1 *A‐D*. According to Pialat and colleagues,^(^ ^30^ ^)^ all measurements were graded by a single operator on a scale from 1 to 5 (G1: no motion artifacts, G5: extreme motion artifacts). Measurements with a grading score of 4 and 5 were regarded non‐evaluable and repeated up to three times.

#### Image processing

After reconstruction respective masks and segmentations for cortical and trabecular bone compartments were created using the manufacturer's standard evaluation protocols (IPL Scanco Module 64‐bit Version V5.16/FE‐v02.02). In brief, the periosteal and endosteal surfaces were automatically defined and segmentations were performed using a Gauss filter (sigma = 0.8, support = 1) and two thresholds (trabecular bone: 320 mg hydroxyapatite [HA]/cm^3^, cortical bone: 450 mg HA/cm^3^). Standard bone density and structural parameters were computed (abbreviations according to^(^ ^31^ ^)^ summarized in Table 1).

**Table 1:** Abbreviations of HR‐pQCT Derived Basic Bone Density and Structural Parameters

Abbreviation	Parameter	Unit of measure
Total vBMD	Total volumetric bone mineral density	mg/cm^3^
Tb vBMD	Trabecular volumetric bone mineral density	mg/cm^3^
Ct vBMD	Cortical volumetric bone mineral density	mg/cm^3^
Tb BV/TV	Trabecular bone volume fraction	%
Tb N	Trabecular number	1/mm
Tb Th	Trabecular thickness	mm
Tb Sp	Trabecular separation	mm
Ct Th	Cortical thickness	mm
Ct Po	Cortical porosity	%
Ct Pm	Cortical perimeter	mm
Ct Po Dm	Cortical pore diameter	mm

Abbreviations according to Bonaretti and colleagues.^(^ ^29^ ^)^

#### Estimation of bone strength and stiffness by homogenized finite element analysis

Each measurement was evaluated for bone stiffness and ultimate load using a standardized and validated nonlinear homogenized finite element pipeline published by Hosseini and colleagues^(^ ^32^ ^)^ and Arias‐Moreno and colleagues^(^ ^33^ ^)^ available on the HRpQCT scanner software (IPLV5.16/FE‐v02.02; Scanco Medical AG). A brief overview follows. Brick elements with eight‐node and 1.7‐mm edge length were created from the downscaled (factor = 28) periosteal contours. Based on vBMD and a fabric tensor based on mean interception length (MIL^(^ ^34^ ^)^), each element was assigned transverse isotropic bone material properties (Young's modulus *ε*_0_ = 19 GPa, shear modulus μ_0_ = 7.9 GPa, compressive strength σ_0_^−^ = 166 MPa, maximum tensile strength *σ*_0_^+^ = 131 GPa, and maximum shear strength τ_0_ = 67 GPa). In the absence of quantitative data regarding human bone material properties in T1DM, the same elastic and postelastic material constants were used for all bones.

For elements containing only cortical bone, isotropic material behavior was assumed. Accordingly, the MIL fabric tensor was fixed to the identity tensor. The used constitutive law is described in detail in Hosseini and colleagues.^(^ ^35^ ^)^ It involves a linear elastic region followed by yielding with a simultaneous buildup of damage and irreversible strains. Displacements of the most distal nodes were constrained in the two in‐plane directions and displacements of the most proximal nodes were constrained in all three degrees of freedom (DOF). A uniform axial compression of 1% strain was applied perpendicular to the most distal surface on a virtual node rigidly coupled to the nodes of the most distal layer. Displacements and reaction forces were measured at the virtual node. Ultimate load was defined to be the maximum recorded force value and stiffness was computed from the force‐displacement curve as the initial slope. All homogenized finite element (hFE) calculations were performed on the XCTII system computer using a single core on an HP Integrity Server rx2800 i4.

### Statistical analysis

Baseline characteristics were described as percentage of participants, or mean ± standard deviation (SD) if normally distributed, and median and interquartile range (IQR) if not. The analysis was performed separately for T1DM and controls. To compare the baseline characteristics, a Mann‐Whitney *U* test was used to test for differences between continuous variables, and a chi square test or a Fisher's exact test for categorical variables.

Multivariable logistic regression analyses were implemented for further analyses of data. Multivariable models were built with each bone score measured by DXA or HRpQCT as dependent variables, and age, sex, body mass index (BMI), and diagnosis (T1DM versus control) as independent variables. We performed a second multivariable analysis in the T1DM patient group discriminating for presence of polyneuropathy measured by vibration perception test. The multivariable models were built using bone parameters as dependent variables and age, gender, BMI, and presence of diabetic neuropathy as independent variables. Data were analyzed using R software14 version 4.0.0 (2020‐04‐24; R Foundation for Statistical Computing, Vienna, Austria.

## Results

### General characteristics of the study population

We recruited 59 patients with T1DM and 77 nondiabetic controls (CO) for this study. Table 2 shows the characteristics of all participants. A *p* value of <0.05 was considered significant. There were no significant differences in age and BMI. Gender distribution was unevenly balanced with more women in the control group (24 T1DM versus 47 CO) and more men in the diabetic group (35 T1DM versus 30 CO). More women in the control group were postmenopausal (*p* = 0.04). Alcohol intake was higher in patients with T1DM (*p* = 0.01). There were no significant differences in fracture prevalence including occult vertebral fractures, nor differences in the prevalence of smoking or daily calcium intake. Participants with T1DM needed significantly more time for the chair rising test (*p* < 0.01) than controls, whereas no difference was observed for the timed up and go test.

**Table 2:** General Characteristics of the Study Population

Characteristic	T1DM (*n* = 59)	Controls (*n* = 77)	*p*
Gender female/male, *n*	24/35	47/30	**0.04**
Age (years), mean ± SD	59.9 ± 9.9	60.9 ± 7.5	0.50
BMI (kg/m^2^), mean ± SD	25.5 ± 3.7	25.3 ± 4.0	0.53
Postmenopausal status, *n*	22	46	**0.04**
Postmenopausal hormone replacement, *n*	2	10	0.10
Smoking (current/past), *n*	9/22	10/25	0.90
Alcohol consumption (U/d), median (IQR)	0.5 (0.0–1.0)	0.1 (0.0–0.5)	**0.01**
Daily calcium intake (mg), mean ± SD	740 ± 349	799 ± 304	0.30
Low traumatic fractures, *n*	0	0	1.00
Past traumatic fractures, *n*	20	22	0.80
Fractures assessed by VFA (*n* = 115), *n*	0	0	1.00
Falls in the last 12 months, *n*	0 (0–1)	0 (0–0.3)	0.44
Timed up and go test (seconds), median (IQR)	6.0 (6.0–8.0)	6.0 (6.0–7.0)	0.90
Chair rise test (seconds), median (IQR)	12.5 (11.0–14.75)	11.0 (10.0–13.0)	**<0.0**

Data are expressed as mean ± SD, median (interquartile range), or numbers (*n*). Significant values are shown in bold. Values of *p* were calculated by chi‐square or Fisher’s exact test in case of dichotomic variables and by Mann‐Whitney test in case of continuous variables.

BMI = body mass index; IQR = interquartile range; VFA = vertebral fracture assessment.

### Diabetes‐related parameters

T1DM patients had a mean disease duration of 37.7 years (Table 3).

**Table 3:** Diabetes‐Related Parameters

Parameter	T1DM (*n* = 59)
Diabetes duration (years), mean ± SD	37.7 ± 9.0
Glycemic control	
HbA1c (%), median (IQR)	6.8 (5.4–7.4)
HbA1c 2 years ago (%) (*n* = 46), median (IQR)	7.1 (6.8–7.8)
HbA1c 5 years ago (%) (*n* = 32), median (IQR)	7.1 (6.8–7.4)
HbA1c 7 years ago (%) (*n* = 17), median (IQR)	6.9 (6.7–7.2)
HbA1c 10 years ago (%) (*n* = 18), median (IQR)	7.0 (6.4–7.5)
Fasting glucose (mmol/L), median (IQR)	8.6 (7.2–11.2)
Hx of hypoglycemia grade II/III, *n*/*N* (%)	31/59 (52.5)
Hypoglycemia grade II/III, past 12 months, *n*/*N* (%)	6/59 (10.2)
Hypoglycemia grade II/III, past 3 months, *n*/*N* (%)	5/59 (8.5)
Insulin treatment	
Mean daily insulin dose (IU), mean ± SD	44.4 ± 20.6
Functional insulin therapy, *n*/*N* (%)	35/59 (59.3)
Conventional basis/bolus therapy, *n*/*N* (%)	24/59 (40.7)
Microvascular and macrovascular complications, *n*/*N* (%)	
Retinopathy	26/59 (44.1)
Nephropathy, defined as microalbuminuria	10/59 (16.9)
Diabetic peripheral neuropathy	22/59 (37.2)
Diabetic foot syndrome	2/59 (3.5)
Presence of any microangiopathy	38/59 (64.4)
Cardiac disease	10/59 (16.9)
Peripheral arterial disease	4/59 (6.8)
More severe disease^a^	22/59 (37.3)

Data are expressed as mean ± SD or median (IQR) or *n*/*N* (%).

Hx = history; IQR = interquartile range; IU = international units; .

^a^ Defined as at least two diabetic microvascular or macrovascular complications.

#### Glycemic control

Median Hba1c level was 6.8%. Long‐term glycemic control was documented by historical HbA1c data: median HbA1c was 7.1% 2 years ago, 7.1% 5 years ago, 6.9% 7 years ago and 7.0% 10 years ago. A total of 31 T1DM patients (52.5%) reported a history of severe hypoglycemia grade II and III.

#### Microvascular and macrovascular complications

A total of 38 diabetic patients (64.4%) had evidence of any microangiopathy. 26 patients (44.1%) suffered with diabetic retinopathy; 10 patients (16.9%) had diabetic nephropathy defined as evidence of microalbuminuria; 22 patients (37.2%) were diagnosed with diabetic peripheral neuropathy. Patients with and without diabetic neuropathy were comparable in terms of diabetes duration, glycemic control, episodes of severe hypoglycemia, and frequency of falls (Table S1).

More severe disease, defined as at least two diabetic microvascular or macrovascular complications, was found in 22 patients (37.2%).

### Biochemical assessment

There were no significant differences in phosphate, iPTH and creatinine levels between T1DM and controls (Table 4). We saw a tendency toward lower 25OH Vitamin D levels in T1DM (*p* = 0.05). Albumin‐corrected calcium was significantly higher in patients with T1DM (*p* = 0.02) but absolute differences were minimal.

**Table 4:** Biochemical Assessment

Parameter	T1DM	Controls	*p*
HbA1c (%)	6.8 (5.4–7.4)	5.2 (4.0–5.4)	**<0.01**
Fasting glucose (mmol/L)	8.6 (7.2–11.2)	5.3 (4.9–5.8)	**<0.01**
Calcium, albumin‐corrected (mmol/L)	2.30 (2.23–2.34)	2.26 (2.20–2.32)	**0.02**
Phosphate (mmol/L)	1.2 (1.0–1.3)	1.1 (1.1–1.3)	0.16
PTH intact (pg/mL)	35.1 (29.2–45.5)	39.5 (31.3–51.7)	0.12
25 OH Vitamin D (nmol/L)	55 (41–78)	65 (54–78)	0.05
Creatinine (μmol/L)	74 (65–88)	73 (68–82)	0.77
Urine calcium (mmol/L)	0.6 (0.3–1.0)	0.7 (0.3–1.2)	0.62
Urine creatinine (mmol/L)	3.5 (2.1–7.3)	3.4 (1.1.‐6.3)	0.2

Data are expressed as median (IQR). Significant values are shown in bold. Values of *p* were calculated by chi‐square or Fisher’s exact test in case of dichotomic variables and by Mann‐Whitney test in case of continuous variables.

IQR = interquartile range.

After adjustment for age, gender, and BMI, serum CTX was significantly decreased in T1DM (*p* < 0.01) whereas there were no significant differences in P1NP and BAP between T1DM and controls (Table 5). CTX was not significantly different between patients with and without diabetic neuropathy in a multivariate regression model adjusted for age, gender, and BMI (estimate, [95% confidence interval]: 0.03, [−0.05, 0.11], *p* = 0.47).

**Table 5:** Comparison of Bone Turnover Markers and DXA Data in Patients With T1DM and Controls Matched by Age, Gender, and BMI

Parameter	Estimate T1DM versus CO	95% CI	*p*
P1NP (ng/mL)	0.47	−7.31, 8.27	0.90
BAP (μg/L)	0.90	−0.78, 2.57	0.29
CTX (ng/mL)	−0.09	−0.15, −0.03	**<0.01**
Lumbar spine BMD (mg/cm^2^)	−0.05	−0.11, −0.001	**0.04**
Lumbar spine *T* score	−0.49	−0.98, 0.002	0.05
Lumbar spine *Z*‐score	−0.49	−0.99, 0.0002	0.05
Total hip BMD (mg/cm^2^)	−0.07	−0.11, −0.03	**<0.001**
Total hip *T*‐score	−0.51	−0.81, −0.21	**<0.01**
Total hip *Z*‐score	−0.49	−0.79, −0.19	**<0.01**
Femoral neck BMD (mg/cm^2^)	−0.42	−0.85, 0.01	0.05
Femoral neck *T*‐score	−0.53	−0.80, −0.26	**<0.01**
Femoral neck *Z*‐score	−0.53	−0.79, −0.27	**<0.01**
Distal radius BMD (mg/cm^2^)	−0.02	−0.04, −0.005	**0.01**
Distal radius *T*‐score	−0.44	−0.86, −0.02	**0.04**
Distal radius *Z*‐score	−0.42	−0.85, 0.01	0.05
TBS	−0.37	−0.07, 0.001	0.06

Values of *p* were calculated by a multivariate linear regression model adjusted for age, gender, and BMI.

TBS = trabecular bone score.

### aBMD at the spine, hip, and distal radius

In patients with T1DM (*n* = 59) we found a significantly lower aBMD at the total hip compared to controls (*n* = 77) (*p* < 0.001) as shown in Table 5. aBMD at the lumbar spine (*p* = 0.04), femoral neck (*p* = 0.05), and distal radius (*p* = 0.01) was lower in patients with T1DM after correction for age, gender, and BMI. aBMD at the total hip did not significantly differ in diabetic patients with and without diabetic peripheral neuropathy in a multivariate regression analysis (estimate, [95% confidence interval]: −0.01, [−0.07, 0.04], *p* = 0.61).

Although there was a trend toward lower TBS in patients with T1DM, findings were not significant. The FRAX scores for hip (*p* < 0.01) and major osteoporotic fractures (*p* = 0.02) were significantly higher among the diabetic group. Two patients with T1DM and none from the control group reached the intervention threshold for bone‐specific medical treatment based on the 10‐year risk of a major osteoporotic fracture in Switzerland as defined by the Swiss Association against Osteoporosis (SVGO).^(^ ^36^ ^)^

### HRpQCT data at the ultradistal tibia and radius

We performed HRpQCT in 51 patients with T1DM and 64 controls. All findings were adjusted for age, gender, and BMI.

#### Ultradistal tibia

##### vBMD, bone microarchitecture, and bone strength in T1DM and controls

We found a significantly reduced cortical thickness (*p* < 0.01) and reduced cortical vBMD (*p* = 0.03) in patients with T1DM compared to controls (Table 6). Although we observed a trend toward lower total vBMD in T1DM, findings were not significant. Trabecular vBMD and trabecular microarchitecture were not significantly altered in T1DM. Bone strength (*p* < 0.01) and bone stiffness (*p* < 0.01) were significantly reduced in T1DM in comparison to their control counterparts.

**Table 6:** Ultradistal Tibia: Comparison of HRpQCT Data in T1DM and Controls and T1DM DN+ and T1DM DN− Matched by Age, Gender, and BMI

Parameter	Estimate T1DM versus CO	95% CI	*p*	Estimate T1DM DN+ versus T1DM DN−	95% CI	*p*
Volumetric density						
Total vBMD (mg/cm^3^)	−14.28	−30.19, 1.62	0.08	−20.96	−46.93, 5.01	0.11
Ct vBMD (mg/cm^3^)	−28.66	−54.38, −2.93	**0.03**	−49.28	−90.04, −8.52	**0.02**
Tb vBMD (mg/cm^3^)	−6.76	−19.88, 6.37	0.31	−1.23	−20.45, 17.98	0.90
Microarchitecture						
Tb BV/TV	−0.01	−0.03, 0.01	0.2	0.005	−0.02, 0.03	0.66
Tb N (1/mm)	−0.02	−0.09, 0.05	0.61	0.05	−0.09, 0.18	0.50
Tb Th (mm)	−0.003	−0.01, 0.004	0.43	−0.004	−0.02, 0.01	0.45
Tb Sp (mm)	0.005	−0.03, 0.04	0.74	−0.04	−0.09, 0.01	0.14
Ct Th (mm)	−0.14	−0.24, −0.05	**<0.01**	−0.11	−0.27, 0.05	0.15
Ct Po (%)	−0.001	−0.004, 0.003	0.65	0.001	−0.003, 0.004	0.73
Ct Pm (mm)	−2.12	−5.13, 0.88	0.16	4.87	−0.96, 10.69	0.10
Ct Po Dm (mm)	−0.005	−0.01, 0.002	0.15	−0.009	−0.02, 0.003	0.14
FE analysis						
Bone stiffness (N/mm)	−8902.3	−14380.5, −3424.2	**<0.01**	−11247.0	−19844.9, −2649.1	**0.01**
Bone strength (N)	−2216.38	−3822.9, −609.8	**<0.01**	−2927.97	−5328.8, −527.1	**0.02**

Data are shown as estimates with a 95% confidence interval. Significant values are shown in bold. Values of *p* were calculated by a multivariate linear regression model adjusted for age, gender, and BMI.

T1DM DN+ = T1DM with diabetic neuropathy; T1DM DN− = T1DM without diabetic neuropathy.

##### vBMD, bone microarchitecture, and bone strength in T1DM with and without neuropathy

We further characterized the diabetes associated cortical bone deficit at the tibia comparing patients with and without diabetic neuropathy (DN) to nondiabetic controls.

**Fig. 2:**
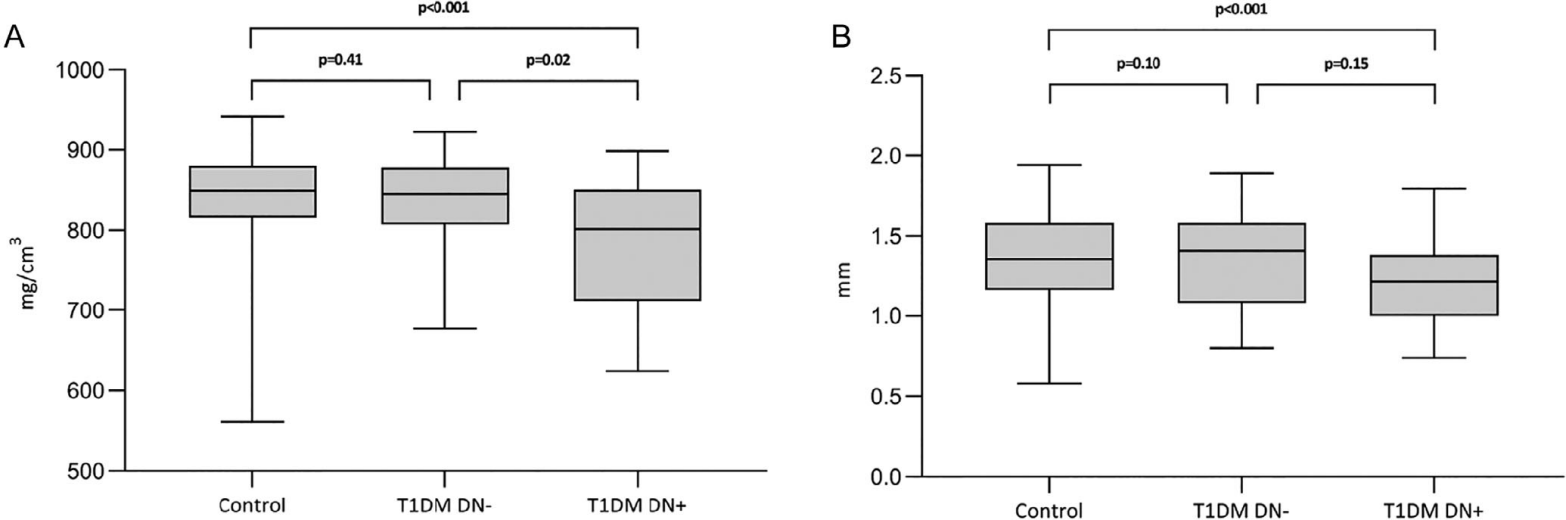
Cortical vBMD (*A*) and cortical thickness (*B*) at the ultradistal tibia in nondiabetic controls and T1DM with and without diabetic neuropathy. Values of *p* were calculated by a multivariate regression model adjusted for age, sex, and BMI. Ct vBMD = cortical vBMD; Ct.Th = cortical thickness; T1DM DN− = T1DM without diabetic neuropathy; T1DM DN+ = T1DM with diabetic neuropathy.

Participants with T1DM and DN (T1DM DN+) showed a significantly reduced cortical vBMD (*p* < 0.01) compared to controls. Cortical vBMD was not significantly different between T1DM without DN (T1DM DN−) and controls (*p* = 0.41). T1DM DN+ had a significantly lower cortical vBMD than T1DM DN− (Fig. 2*A*).

Findings for cortical thickness followed a similar pattern with reduced cortical thickness (*p* < 0.01) in T1DM DN+ as compared to controls. Cortical thickness was not significantly different in T1DM DN− compared to controls, and T1DM DN+ compared to T1DM DN−. (Fig. 2*B*).

We compared cortical vBMD in T1DM with any microangiopathy to T1DM without microangiopathy: Cortical vBMD at the tibia was not significantly lower in patients with any microangiopathy (estimate, [95% confidence interval]: −34.82, [−75.84, 6.20], *p* = 0.09). Similarly, patients with more severe disease, defined as a diagnosis of at least two microvascular or macrovascular complications, did not show a significantly decreased cortical vBMD at the tibia (estimate, [95% confidence interval]: −35.45, [−76.19, 5.29], *p* = 0.08).

**Fig. 3:**
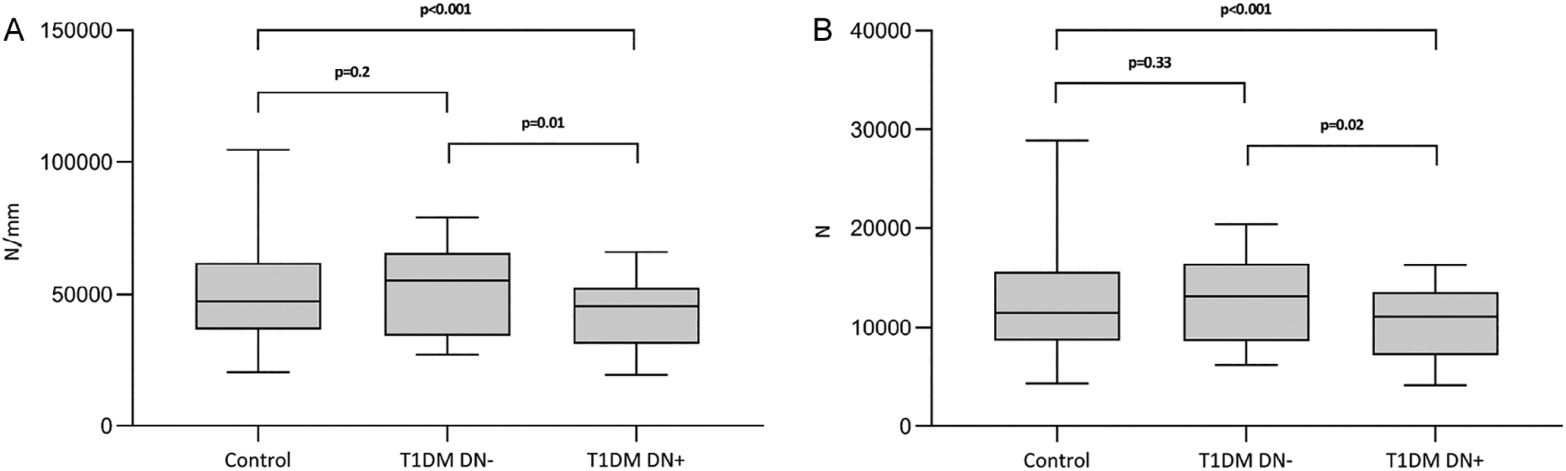
Bone stiffness (*A*) and bone strength (*B*) at the ultradistal tibia in nondiabetic controls and T1DM with and without diabetic neuropathy. Values of *p* were calculated by a multivariate regression model adjusted for age, sex, and BMI. T1DM DN− = T1DM without diabetic neuropathy; T1DM DN+ = T1DM with diabetic neuropathy.

T1DM DN+ had a significantly lower estimated bone strength (*p* = 0.02) and bone stiffness (*p* = 0.01) compared to T1DM DN−. When comparing T1DM DN− with controls there was no significant difference in bone strength or stiffness. T1DM DN+ showed a highly significant reduction in bone stiffness (*p* < 0.001) and bone strength (*p* < 0.001) compared to nondiabetic controls. (Fig. 3*A*,*B*).

#### Ultradistal radius

No significant differences between T1DM and controls were observed for total, cortical, and trabecular volumetric density at the ultradistal radius (Table 7). Except for a significantly lower cortical perimeter (*p* < 0.01) in T1DM, none of the other microarchitectural parameters was significantly different between T1DM and controls. Although estimates of bones stiffness and bone strength were lower in patients with T1DM compared to controls, findings were not significant.

**Table 7:** Ultradistal Radius: Comparison of HRpQCT Data in T1DM and Controls and T1DM DN+ and T1DM DN− Matched by Age, Gender, and BMI

Parameter	Estimate T1DM versus CO	95% CI	*p*	Estimate T1DM DN+ versus T1DM DN−	95% CI	*p*
Volumetric density						
Total vBMD (mg/cm^3^)	0.25	−20.12, 20.62	0.98	−35.30	−71.44, 0.85	0.05
Ct vBMD (mg/cm^3^)	3.28	−12.72, 19.29	0.68	−39.14	−97.22, 18.94	0.18
Tb vBMD (mg/cm^3^)	−4.49	−17.87, 8.88	0.51	−6.30	−25.38, 12.79	0.50
Microarchitecture						
Tb BV/TV	−0,01	−0.03, 0.01	0.32	−0.01	−0.03, 0.02	0.55
Tb N (1/mm)	0.005	−0.01, 0.02	0.65	−0.002	−0.04, 0.04	0.90
Tb Th (mm)	0.0001	−0.01, 0.01	0.97	0.001	−0.01, 0.01	0.91
Tb Sp (mm)	0.01	−0.01, 0.04	0.49	−0.02	−0.11, 0.08	0.74
Ct Th (mm)	−0.02	−0.1, 0.06	0.61	−0,12	−0.27, 0.03	0.1
Ct Po (%)	−0.0002	−0.0001, 0.0004	0.80	0.003	−0.001, 0.01	**<0.01**
Ct Pm (mm)	−3.6	−5.63, −1.58	**<0.01**	4.87	1.81, 7.89	**<0.01**
Ct Po Dm (mm)	−0.003	−0.012, 0.006	0.50	0.02	0.006, 0.034	**<0.01**
FE analysis						
Bone stiffness (N/mm)	−2163.6	−5624.1, 1296.5	0.22	−1907.1	−7549.6, 3735.4	0.50
Bone strength (N)	−467.17	−1118.6, 184.2	0.16	−267.0	−1277.4, 725.5	0.58

Data are shown as estimates with a 95% confidence interval. Significant values are shown in bold. Values of *p* were calculated by a multivariate linear regression model adjusted for age, gender and BMI.

T1DM DN+ = T1DM with diabetic neuropathy; T1DM DN− = T1DM without diabetic neuropathy.

## Discussion

This is the first study to assess bone mineral density, bone microarchitecture, biochemical, and estimated biomechanical bone parameters in patients with long‐standing, well‐controlled T1DM. Compared to nondiabetic controls we observed a reduced aBMD at all measured sites, low CTX, a marker of bone resorption, and a cortical bone deficit at the ultradistal tibia with impaired bone strength and bone stiffness as modeled by hFE analysis. Both the reduced cortical vBMD and lower cortical thickness as well as the significantly altered biomechanical parameters were dependent on the presence of diabetic peripheral neuropathy.

In our cohort of patients with a mean age of 60 years with excellent long‐term glycemic control, we found a highly significant aBMD reduction at the total hip as well as a reduced aBMD at the femoral neck, lumbar spine, and distal radius. A recent, large study comparing aBMD in children and adults with T1DM to healthy controls did not reveal aBMD differences across age groups, except for a reduced aBMD in postmenopausal women at the spine, femoral neck, and total hip.^(^ ^37^ ^)^ However, in line with our findings, most studies in adult T1DM show a reduction in aBMD^(^ ^38^ ^)^ with a meta‐analysis reporting an average decrease of −22% in spine BMD and of −37% in hip *Z*‐score.^(^ ^7^ ^)^

T1DM has been reported to be a state of low bone turnover^(^ ^39^ ^)^ with reduced bone formation and bone resorption as a potential determinant of altered bone microstructure. We found significantly lower CTX in our cohort of long‐standing, nonfracturing T1DM. Bone resorption assessed by CTX might underestimate actual bone resorption as enzymatic collagen cross‐linking is impaired in diabetes^(^ ^40^ ^)^ and CTX assay measures cross‐linked telopeptides.^(^ ^38^ ^)^ Interestingly, we did not see any significant differences in P1NP or BAP between T1DM and controls. In a meta‐analysis Hygum and colleagues showed that P1NP was not significantly lower in T1DM but due to paucity of data available only two studies were evaluated.^(^ ^41^ ^)^ However, low bone formation in T1DM was confirmed on the basis of decreased osteocalcin levels. The mineralization marker BAP was not significantly different between patients with diabetes and controls.^(^ ^42^ ^)^ The gold standard for estimation of bone turnover in diabetes is bone tissue biopsy: In 1995, Krakauer and colleagues^(^ ^42^ ^)^ performed bone biopsies in two male patients with long‐standing T1DM and found low bone turnover, yet no data is available regarding glycemic control. Armas and colleagues^(^ ^43^ ^)^ reported no differences in bone turnover between T1DM and controls in a large histomorphometry study in patients with well‐controlled T1DM (median HbA1c 6.8%) and a disease duration of 15 years. Whether glycemic control may influence bone turnover in long‐standing T1DM remains to be elucidated. Our findings indicate that long‐standing, well‐controlled diabetes is associated with low bone resorption and unaltered bone formation demonstrated by P1NP and BAP but further research is warranted.

In line with the imbalanced remodeling, we observed microarchitectural changes with a lower cortical vBMD and lower cortical thickness at the ultradistal tibia as measured by HRpQCT. In contrast, no such changes were seen at the ultradistal radius. HRpQCT measurement at the ultradistal radius is more prone to motion artefacts than assessment at the tibia, which may have compromised our data. Yet there is compelling evidence indicating a differing fracture risk at the radius and tibia in T1DM: In a recent meta‐analysis Wang and colleagues^(^ ^44^ ^)^ reported a significant increase in ankle fractures in patients with T1DM (risk ratio [RR] 1.71; 95% CI [1.06, 2.78]; *p* = 0.029), which was more pronounced than in T2DM. Vilaca and colleagues^(^ ^45^ ^)^ showed that diabetes is associated with an increase in the risk of ankle fractures and a decrease in wrist fractures, but most data were obtained from T2DM.

We speculate that our discrepant findings at the tibia and radius may be related to the fact that the ultradistal tibia is a weight‐bearing bone, unlike the radius. Bone adjusts to loading by adaptation of bone mass and microarchitecture.^(^ ^46^ ^)^ Our diabetic cohort with a mean age of 60 years showed worse chair rise performance potentially reflecting early changes in functional mobility. We did not observe any difference in timed up and go test between T1DM and controls. In contrast to the chair rising test, which apart from assessing balance and coordination mainly reflects muscular power of the lower limbs,^(^ ^47^ ^)^ timed up and go performance does not focus on a single motor task but reflects performance of a variety of daily life activities.^(^ ^48^ ^)^ We speculate that decreased functional mobility with impairment of lower limb strength might alter mechanical loading and lead to bone loss predominantly affecting weight‐bearing bones.

Estimated bone strength and bone stiffness at the tibia were strongly compromised in T1DM. Bone strength is determined by both bone mass and bone quality and is an important determinant of fracture risk.^(^ ^49^ ^)^ Ultimately, the cortical bone deficit could increase fracture risk at the distal tibia.

It has been reported that long‐term hyperglycemia in diabetes favors the accumulation of advanced glycation end‐products (AGEs) causing nonenzymatic cross‐linking of type I collagen.^(^ ^50^ ^)^ which seems to impair bone tissue toughness in vitro and in vivo.^(^ ^51^ ^)^ Despite the reported degradation of tissue‐level material properties in two mouse models for diabetes, there was no such evidence in patients with T1DM in a review by Lekkala and colleagues.^(^ ^52^ ^)^ However, a recent study reported a less than 5% decrease in the median of bone material strength index (BMSi) measured with a reference point indenter (OsteoProbe) in a small cohort of males with T1DM compared to controls.^(^ ^53^ ^)^ Interestingly, this finding is consistent with the reduction of cortical vBMD observed in our cohort with long‐standing T1DM. If the observed decrease in tissue material properties that constitute a necessary input for hFE analysis was confirmed, it would imply an overestimation of bone strength for the T1DM group by a similar amount of about 5%. Although this confirmation will require further research, it would reinforce the substantial reduction in bone strength at the ultradistal tibial observed in T1DM that is mainly attributed to thinning of the cortex.

Cortical bone loss is associated with exposure of intracortical surfaces; incompletely refilled excavated sites increase in number and coalesce leading to increased cortical porosity.^(^ ^54^ ^)^ T2DM has been widely accepted to be associated with increased cortical porosity.^(^ ^55^ ^)^ Data for T1DM is less clear; recently Vilaca and colleagues^(^ ^24^ ^)^ showed a higher cortical porosity in T1DM with diabetic neuropathy. Although we found a decreased cortical vBMD and cortical thickness, we were unable to see any significant differences in cortical porosity between T1DM and controls at the radius or tibia. Results for cortical porosity were highly variable in our population and hence difficult to interpret. Image quality has a major impact on assessment of cortical porosity. Cortical porosity may also have been underestimated by HRpQCT: a study using electron microscopy in women aged 63 years showed that intracortical remodeling by cavitation may leave cortical remnants that were falsely identified as trabecula by HRpQCT.^(^ ^54^ ^)^

Our microarchitectural findings with a lower cortical vBMD, cortical thickness, and reduced estimated bone strength and bone stiffness at the tibia were all dependent on the presence of diabetic neuropathy. There was no significant association with other microvascular complications. Hip aBMD and CTX were not significantly altered by the presence of diabetic neuropathy.

Diabetic neuropathy is one of the most common complications of diabetes with 54% of T1DM developing diabetic neuropathy over the course of their life.^(^ ^56^ ^)^ The prevalence increases with diabetes duration, which is a major predictor of diabetic neuropathy.^(^ ^56^ ^)^

The cross‐sectional study design does not allow to draw conclusions about causality, but nevertheless the association of diabetic neuropathy and a cortical bone deficit with reduced estimated bone strength at the tibia only is compelling. Recently, a large prospective study confirmed that bone microstructural changes independently contribute to fracture risk.^(^ ^57^ ^)^ A recent meta‐analysis suggests that patients with diabetic neuropathy have a significantly increased risk of developing osteoporosis and fragility fractures.^(^ ^58^ ^)^ Different potential mechanisms are being discussed, including a neural dysregulation of vascular supply to the bone (neurovascular hypothesis), gait changes leading to altered mechanical loading with repetitive microtrauma (neurotraumatic hypothesis), or an impaired local neurotransmitter release (neurotrophic hypothesis).^(^ ^58^, ^59^ ^)^

Our finding of an impaired cortical microarchitecture at the ultradistal tibia in T1DM is in contrast to previous data^(^ ^20^, ^21^, ^43^, ^60^ ^)^ showing mainly differences in the trabecular compartment. Devaraja and colleagues^(^ ^61^ ^)^ observed an altered trabecular microarchitecture with reduced bone strength at the ultradistal radius and tibia in adolescents with T1DM. In a subanalysis of their study looking at patients with a diabetes duration >2 years, the reduction in load‐bearing at the tibia disappeared. Patients with early manifestation of T1DM present with a transiently impaired bone development^(^ ^62^ ^)^ that normalizes over time.

Shanbhogue and colleagues^(^ ^20^ ^)^ found alterations in the cortical and trabecular compartment in T1DM with microangiopathy only, another recent case‐control study showed changes in the trabecular compartment in T1DM, but no association between microvascular complications and bone microarchitecture.^(^ ^19^ ^)^ However, most of the studies investigating bone quality in T1DM were performed in patients with an average exposure to hyperglycemia of 16 years^(^ ^25^‐diabetes duration was relatively short in comparison to our study. Diabetic complications and specifically diabetic neuropathy may not have yet manifested its effects on bone at this time, suggesting it may have been too early to see a cortical bone deficit.^(^ ^25^ ^)^

It has been proposed that diabetes with microangiopathy is associated with accelerated bone aging.^(^ ^63^ ^)^ The majority of age‐related bone loss at the appendicular skeleton occurs in the cortical compartment,^(^ ^64^, ^65^ ^)^ where we observed changes in our diabetic cohort with a mean age of 60 years. Both our microarchitectural findings and densitometric data with a prominent aBMD deficit at the total hip point to a cortical bone deficit. Whether long‐standing T1DM with diabetic neuropathy might precipitate age‐related bone loss remains to be elucidated.

This present study's findings should be interpreted within the context of its strengths and limitations. Although the comprehensive evaluation of a large cohort of patients with long‐standing T1DM with HbA1c data over the past 10 years is a strength, its overall good glycemic control is a limitation. The homogeneity in diabetes control does not allow for further assessment of any exposure‐response relationship of glycemic control on microstructural changes independent of microvascular disease. Furthermore, we were not able to obtain data on the time of diagnosis of diabetic neuropathy. Data on the frequency of falls and hypoglycemia were self‐reported and may have been underrepresented.

In conclusion, long‐standing, well‐controlled T1DM is associated with a decreased aBMD, low bone turnover, and compromised cortical bone at the ultradistal tibia with reduced estimates of bone strength and stiffness. Both the impaired cortical parameters and the altered estimated biomechanical properties at the tibia are dependent on the presence of diabetic neuropathy. Further research is warranted to evaluate whether these structural changes and specifically the presence of diabetic neuropathy can explain the increased fracture risk in T1DM.

## Author Contributions

**Lilian Sewing:** Investigation; methodology; writing – original draft. **Laura Potasso:** Formal analysis; software. **Sandra Baumann:** Investigation. **Denis Schenk:** Data curation; investigation; software. **Furkan Gazozcu:** Investigation. **Kurt Lippuner:** Resources; writing – review and editing. **Marius Kraenzlin:** Resources. **Philippe K. Zysset:** Data curation; resources; software; writing – review and editing. **Christian Meier:** Conceptualization; funding acquisition; methodology; resources; supervision; writing – review and editing.

## Conflict of Interest

All authors state that they have no conflicts of interest with respect to the submitted manuscript.

### Peer Review

The peer review history for this article is available at https://publons.com/publon/10.1002/jbmr.4517.

## Supporting information


**Supplementary Table S1** Diabetes related parameters in T1DM with and without diabetic neuropathy.

## Data Availability

The data that support the findings of this study are openly available in “figshare” at https://doi.org/10.6084/m9.figshare.16608316
