# Supplementary material for: Bone Microarchitecture and Strength in Long‐Standing Type 1 Diabetes
Source: J Bone Miner Res. 2022 Mar 8;37(5):837–47. doi: 10.1002/jbmr.4517 (PMC9313576; doi:10.1002/jbmr.4517)
Supplement: Supplementary file 1 — Supplementary Table S1 Diabetes related parameters in T1DM with and without diabetic neuropathy. [file JBMR-37-837-s001.docx]

Supplementary Table 1: Diabetes related parameters in T1DM with and without diabetic neuropathy

|  | T1DM DN+ (n=22) | T1DM DN- (n=37) | p |
| --- | --- | --- | --- |
| Diabetes duration, years | 37.7 ± 9.9 | 37.1 ± 8.5 | 0.70 |
| HbA1c, % | 7.1 (6.9-7.9) | 7.2 (6.8-8.0) | 0.85 |
| Hx of Hypoglycaemia grade II/III n/N (%) | 11/22 (50.0) | 18/37 (48.6) | 1.0 |
| Hypoglycaemia grade II/III, past 12 months, n/N (%) | 1/22 (4.5) | 2/37 (5.4) | 1.0 |
| Falls in the last 12 months (n) | 0 (0-1) | 0 (0-0) | 0.08 |

Data are expressed as mean± SD or median (interquartile range). Significant values are shown in bold. p- values are calculated by chi-square or Fisher exact test in case of dichotomic variables and by Mann-Whitney test in case of continuous variables

Abbreviations: T1DM DN+: T1DM with diabetic neuropathy, T1DM DN-: T1DM without diabetic neuropathy
